# Recent State and Challenges in Spectroelectrochemistry with Its Applications in Microfluidics

**DOI:** 10.3390/mi14030667

**Published:** 2023-03-17

**Authors:** Zhenglong Li, Charmi Chande, Yu-Hsuan Cheng, Sagnik Basuray

**Affiliations:** 1 Department of Chemical and Materials Engineering, New Jersey Institute of Technology, Newark, NJ 07102, USA; 2 Department of Biomedical Engineering, New Jersey Institute of Technology, Newark, NJ 07102, USA

**Keywords:** spectroelectrochemistry, ultraviolet-visible SEC, surface-enhanced Raman spectroscopy SEC, nuclear magnetic resonance SEC, dark-field microscopy SEC, microfluidics

## Abstract

This review paper presents the recent developments in spectroelectrochemical (SEC) technologies. The coupling of spectroscopy and electrochemistry enables SEC to do a detailed and comprehensive study of the electron transfer kinetics and vibrational spectroscopic fingerprint of analytes during electrochemical reactions. Though SEC is a promising technique, the usage of SEC techniques is still limited. Therefore, enough publicity for SEC is required, considering the promising potential in the analysis fields. Unlike previously published review papers primarily focused on the relatively frequently used SEC techniques (ultraviolet-visible SEC and surface-enhanced Raman spectroscopy SEC), the two not-frequently used but promising techniques (nuclear magnetic resonance SEC and dark-field microscopy SEC) have also been studied in detail. This review paper not only focuses on the applications of each SEC method but also details their primary working mechanism. In short, this paper summarizes each SEC technique’s working principles, current applications, challenges encountered, and future development directions. In addition, each SEC technique’s applicative research directions are detailed and compared in this review work. Furthermore, integrating SEC techniques into microfluidics is becoming a trend in minimized analysis devices. Therefore, the usage of SEC techniques in microfluidics is discussed.

## 1. Introduction

**Figure 1:**
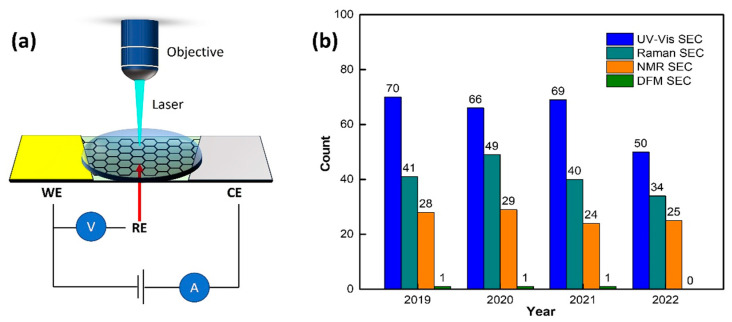
(**a**) Schematic diagram of a combination of electrochemistry and spectroscopy. (**b**) Histograms of statistical results for different spectroelectrochemistry (SEC)-related publications over the last six years. Relevant numbers are obtained from the website of the web of science by searching for “Ultraviolet–visible or UV-Vis; Spectroelectrochemistry”, “Raman spectroscopy; Spectroelectrochemistry”, “Nuclear Magnetic Resonance or NMR; Spectroelectrochemistry”, “Dark field microscopy or DFM; Spectroelectrochemistry.”.

Since the coupling of spectroscopy and electrochemistry (hereafter spectroelectrochemistry (SEC)) can provide a detailed and comprehensive study of the electron transfer kinetics and analytes’ structural information during the electrochemical process. SEC is attracting intensive interest for various research in analytical fields, ranging from biology [1] to chemistry [2], material engineering [3,4], and others [5]. The schematic diagrams of the SEC technique are shown in Figure 1a. Electrochemical techniques, such as cyclic voltammetry (CV), differential pulse voltammetry (DPV), or electrochemical impedance spectroscopy (EIS), have been employed in SEC techniques [6,7]. Similarly, ultraviolet-visible (UV-Vis), Raman/surface-enhanced Raman spectroscopy (SERS), and nuclear magnetic resonance (NMR) are frequently used spectroscopy techniques. Therefore, SEC techniques have incredible versatility because multiple electrochemical methods are available and different spectral regions can be analyzed depending on the system under study, and the desired information to be obtained [8,9,10]. SEC techniques have been used to determine small structural changes and tiny luminescent responses [6,11]. Some of the examples include comprehending the electron transfer kinetics between the electrode and different electrolyte matrices [12], mass transport [13], and redox events for analytes and nanoparticles (NPs) [13,14].

The SEC “family” is continually expanding, including techniques such as dark-field microscopy SEC (DFM SEC), and nuclear magnetic resonance SEC (NMR SEC)) [2,6,11,14,15,16]. The past few decades have witnessed different SEC-combined techniques appearing in various analytical fields. Figure 1b shows articles on the relevant techniques in the past four years. However, even for the two relatively mature combinations of UV-Vis SEC and Raman SEC, relevant published work still needs to be expanded, not to mention the use of NMR SEC and DFM SEC. The development of SEC techniques is still a severe challenge due to the lack of widespread publicity. For this purpose, the recent past few years’ developments in SEC techniques, namely, UV-Vis SEC, Raman SEC, NMR SEC, and DFM SEC, are discussed in this review work. Among the SEC techniques, UV-Vis SEC and Raman SEC are the two most widely used SEC technologies; therefore, it is necessary to know their latest research trends [17,18,19,20,21,22,23]. As limited papers are available on NMR SEC and DFM SEC, this has further limited these two promising SEC technologies’ adoptions. A discussion of these two techniques is essential. Herein, the present review paper is structured by discussing each technique’s basic working principle, and then the current state of the art in the field. Recently, the application of SEC technology in microfluidics has received increasing attention. Therefore, the last section of this article presents the development of combined SEC and microfluidics technology. For SEC techniques, the advantages/disadvantages of them for analysis applications and their future development directions/perspectives are discussed in the summary and outlook sections. Through this review work, we hope more people, whether established researchers or beginners, will be able to see, understand, and use these SEC technologies in their respective research fields.

## 2. Ultraviolet-Visible SEC (UV-Vis SEC)

As stated above, the most commonly used SEC setups are UV-Vis SEC and Raman SEC [24]. UV-Vis SEC is a powerful hybrid technology that allows the researcher to obtain electrochemical and spectroscopic responses simultaneously. UV-Vis SEC, the oldest SEC technique, was introduced in 1964 by Kuwana [25]. In this original work, a tin oxide-coated optically transparent electrode (OTE) platform was used as a WE to understand the mechanism of the oxidation process of *o*-toluidine (C_14_H_16_N_2_) and the absorption features of the electrooxidation products. Subsequently, the UV-Vis SEC approach was used for phenazine detection and exploration of its redox characteristics [26]. Pavel et al. used this technique for the rapid determination of the optical and redox properties of the [Zn_2_(NDC)_2_(DPNI)]^0/−/2−/^metal–organic framework [27].

**Figure 2:**
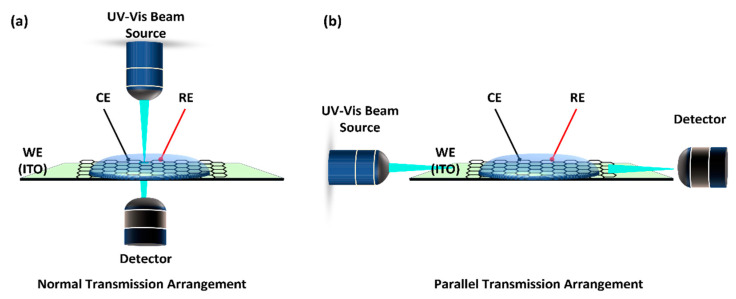
Schematic diagram of two different UV-Vis SEC arrangements. (**a**) Normal transmission arrangement, and (**b**) parallel transmission arrangement.

Based on the arrangement of the light source, the UV-Vis SEC can be roughly divided into two categories, that is, the normal transmission arrangement (Figure 2a) and parallel transmission arrangement (Figure 2b). When the light beam travels perpendicular to the WE surface in the normal configuration, it collects information about the solution and the WE. However, only the solution is sampled when the light beam is parallel to the WE (parallel transmission arrangement) [28]. For the normal transmission arrangement, since the light source needs to penetrate though the analyte solution and the WE, therefore, in this case, OTEs are fundamental for the success of the UV-Vis SEC and a topic of intense research. This drastically reduces the number of WE that can be used. On the other hand, a perfect but difficult alignment of the light beams is required in the parallel configuration. However, in practice, this means that many different parts must be assembled to carry out a single experiment [19].

### 2.1. OTEs in UV-Vis SEC

OTEs are used for a broad range of applications stemming from the fundamental investigation of electron transfer mechanisms to mature applied daily applications, especially in the field of photovoltaics and thin film transistors (TFT) [29,30,31]. At present, the commonly used OTEs are metal oxide films (e.g., indium tin oxide (ITO), fluorine-doped tin oxide (FTO)); thin metal films (e.g., gold (Au), platinum (Pt)); and carbon-based OTEs (e.g., graphene, carbon nanotube, glassy carbon), deposited on borosilicate or quartz glass substrates. Table 1 summarizes the employment of different OTEs in recent applications of UV-Vis SEC.

The most recent studies have concentrated on using carbon-based OTEs [8,32,33,34]. Carbon-based OTEs offer compelling advantages over traditional metal or metal oxide OTEs. These have easy accessibility, excellent chemical inertness, high electrical conductivity, a wide electrochemical potential window, versatile preparation methods, and the simplicity of surface modifications [35,36]. However, carbon-based OTEs also suffer from some disadvantages such as (i) weak adhesion between the substrate and carbon nanomaterials [37,38,39], (ii) low production capacity [40], and (iii) their surfaces may possess a variety of functional groups since the nature of their surfaces can affect their electrochemical performance [41,42,43,44].

**Table 1:** A brief comparison of different OTEs.

Ref.	Cell Characteristics	Research Topic	Method Advantage	Publish Time
[45]	WE: Zinc oxide (ZnO, *Φ* = 6 mm); CE: Pt disk (2.56 mm^2^ surface area); RE: Ag/AgCl	Methylene blue detection	Good optical transparency and electrical conductivity for ZnO OTE; Robust chemical stability; Wide potential window (−1.0 to 1.8 V)	2020
[46]	WE: Indium tin oxide (ITO, 9 mm × 60 mm); CE: Pt wire; RE: Ag/Ag^+^	Substituent effects of H-3Cz, P-3Cz, and E-3Cz during electropolymerization	Exceptional sensitivity; Simultaneous track ionic species and mass change during electropolymerization	2021
[47]	WE: Fluorine-doped tin oxide (FTO) decorated with AgNPs; CE: Pt plate (10 × 20 mm^2^); RE: Ag/AgCl	Monitor of Ampyra level	High sensitivity and selectivity; Low detection limit (~6 µmol/L)	2020
[48]	WE: Free-standing single wall carbon nanotube (SWCNT) film; CE: Pt wire; RE: Ag/AgCl	The electrochemical process of Ferrocenemethanol and Hexacyanoferrate (II), Dopamine (DA) oxidation	Rapid and facile fabrication; Good transparency and conductivity; High reproducibility; Low cost	2016

WE: working electrode; CE: counter electrode; RE: reference electrode; H-3Cz: 9ʹH-9,3′:6ʹ,9′′-tercarbazole; P-3Cz: 3,6-bis(*N-carbazolyl*)-*N*-phenylcarbazole; E-3Cz: 3,6-bis(*N-carbazolyl*)-*N*-ethylcarbazole.

- ITO film has been one of the most used metal oxides OTEs. The thin film layer is typically sputter-coated on a glass substrate [49]. However, because of the less negative inert potential window (i.e., −0.45 to +1.92V vs. reversible hydrogen electrode (RHE) in 0.1M NaOH), this material can only be used in a relatively small range of potentials [50]. In addition, further widespread use may be limited by the cost, brittleness, and scarcity of indium [51]. FTO is another representative metal oxide OTE. Like ITO, FTO also faces the problem of a less negative inert potential window (i.e., −0.51 to 1.73V vs. RHE in 0.1 M NaOH) [50].
- Thin metal films have been widely reported and proven. Especially, sputtered Au film is an appropriate candidate for the thin film electrode material because of its good conductivity, enough transparency, and very low chemical reactivity. Additionally, the electrochemical behavior of Au has been studied extensively [52,53], and thus it may be easier to predict and understand the behavior of an Au electrode. However, Au may not be an ideal electrode material for electrochemical studies that require high potentials due to the corresponding Au oxidation [50].
- Recently, the most reported studies concentrated on using carbon-based OTEs, as stated in Table 1. Carbon-based OTEs offer several advantages over traditional metal and metal oxide OTEs, including easy accessibility, excellent chemical inertness, high electrical conductivity, a wide electrochemical potential window, versatile preparation methods, and the simplicity of surface modifications [35,36]. Unlike ITO and FTO, which have a rapid decrease in the transparency for wavelengths shorter than ~350 nm, carbon-based OTEs can exhibit enough optical transparency over a broader frequency range [35,54]. Although there are also many problems in carbon-based OTEs, such as (i) weak adhesion between the substrate and carbon nanomaterials; (ii) problems related to massive production [40]; and (iii) surface-preparation-dependent-electrochemical performance, the use of carbon-based OTEs is becoming more and more popular.

### 2.2. Applications of UV-Vis SEC

**Figure 3:**
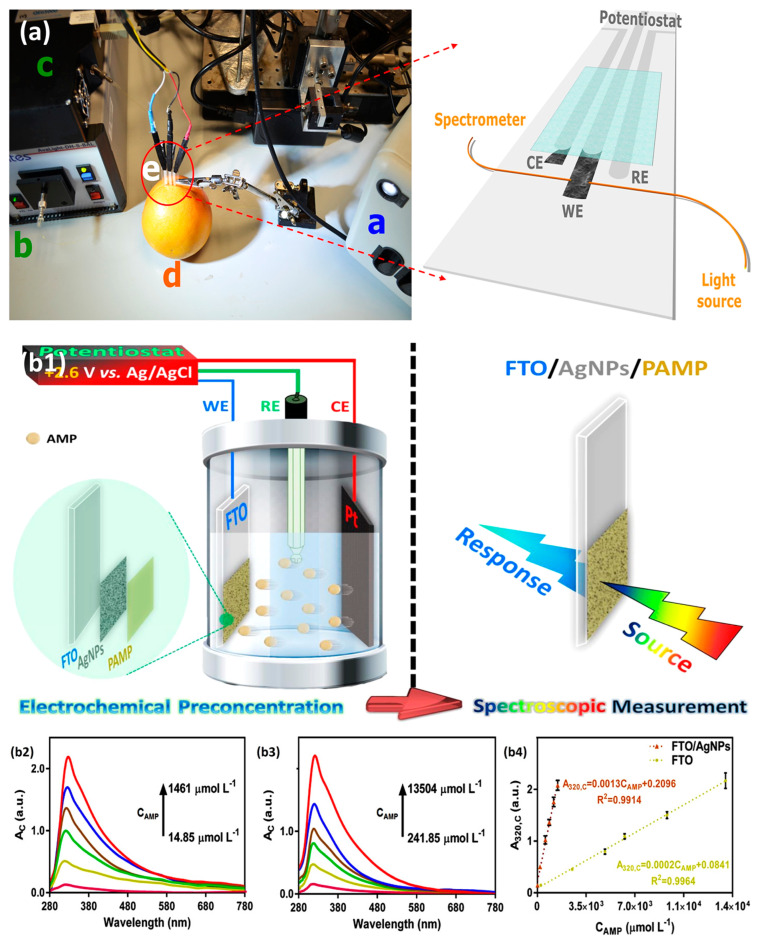
(**a**) Device placed inside the grapefruit to perform an SEC measurement. Setup only requires a potentiostat, light source, spectrometer, grapefruit, and SEC device. Reprinted from [10], with permission from ACS Publications. (**b1**) Schematic illustration of the sensing procedure developed to determine ampyra (AMP). The background-corrected spectra of the (**b2**) FTO/AgNPs and (**b3**) FTO at various concentrations of AMP. (**b4**) The corresponding relationships between the A_320_ and concentrations of AMP. Reprinted from [47], with permission from Elsevier.

It is well-known that UV-Vis SEC has been applied in many fields, for example, in electron transfer processes [55], solar cells [31,56], memory devices [57], and the determination of compounds of biological interest [10,58,59,60]. A detailed summary of the chosen examples’ electrode configurations and light arrangements is given in Table 2. As presented in Figure 3a, an in situ UV-Vis SEC technique interrogating a three-electrode configuration was demonstrated by Jesus et al. to direct the determination of ascorbic acid (AA) in a grapefruit [10]. In this case, the three-electrode cell (a working electrode (WE), a counter electrode (CE) of SWCNTs, and a silver/silver chloride (Ag/AgCl) reference electrode (RE)) was directly placed inside the grapefruit without any pretreatment. Using the electrochemical method (oxidation of AA at +0.90 V), the concentration of AA was found to be [1.99 ± 0.14] × 10^−3^ M. While using the spectroscopic method (UV-Vis), they found the concentration to be [2.06 ± 0.11] × 10^−3^ M. One interesting point of this work that needs elaboration is the preparation of the SWCNT electrodes. First, the SWCNT dispersion was filtered, and subsequently, the SWCNT film was press-transferred on a polyethylene terephthalate (PET) sheet using a stencil with a custom design. As a result, the excellent reproducibility of the SWCNT electrodes was demonstrated. Herein, the light source was paralleled with respect to the WE surface, and the first 100 µm of the solution adjacent to the SWCNT WE surface was recorded for further optical analysis.

Electrochemical preconcentration is one of the most frequently discussed preconcentration techniques at a controlled potential [61,62,63,64]. Hybrid SEC techniques also often involve this method to assist the goal of quantifying ultra-trace aqueous target analytes [47,65]. Such as this latest published paper by Arash et al., in this work, the monitor of a potassium-channel blocking agent, ampyra (AMP), was studied. As shown in Figure 3(b1), a glass cuvette equipped with an FTO transparent WE, a Pt plate (10 × 20 mm^2^) CE, and an Ag/AgCl RE was employed in this work. The light beam transmitted through the WE and arrived at the diode array detector (~1 cm light path). The information on the light intensity located at 320 nm (related to AMP) was recorded and shown in Figure 3(b2–b4). The AgNP decorated FTO WE can show an exceptionally low detection limit of ~5.77 µmol/L AMP, which is satisfactory for quantifying the AMP in commercial tablets.

**Table 2:** Applications summary of UV-Vis SEC, SERS SEC, NMR SEC, and DFM SEC in different research fields.

SEC	Ref.	Electrochemistry	Electrode Configuration	Research Studied	Highlights	Weak Points
		Spectroscopy				
UV-Vis SEC	[65]	CV	WE: Glassy carbon foil or SWCNT; CE: Pt wire; RE: Ag/AgCl	Oxidation of dopamine; Electropolymerization of EDOT	Excellent compatibility to form bidimensional SEC (UV-Vis and Raman); Multiple responses; Wide versatility	Complicated electrochemical structure
		Parallel arrangement				
	[32]	CV	WE/CE: SWCNT; RE: Ag wire	Quantitative resolution of CAT/DA and DA/EP mixtures	High reproducibility; Low cost; Small volume usage of sample solution	Non-ideal *LOD*
		Parallel arrangement				
	[66]	CV, CA, LSV	WE: FTO; CE: Pt foil; RE: Ag/AgCl	Vitamin B_12_ as an OER catalyst	Dual monitoring strategy; High precision and sensitivity	High requirement on the compatibility between different techniques
		Normal arrangement				
	[67]	CV, LSV	WE: Carbon disc; CE: Carbon; RE: Ag wire	Determination of Isoprenaline	Screen-printed electrode; Low cost; Easy-to-use; Longer optical path length; Small volume usage of sample solution	Non-ideal *LOD*; Need pretreatment on the sample
		Parallel arrangement				

SERS SEC	[24]	CV, LSV	WE: AgNPs decorated Ag; CE: Carbon; RE: Ag wire	Quantification of [Fe(CN)_6_]^3−^ and [Ru(bpy)_3_]^2+^	High sensitivity; Real-time; Cost-effective; Low *LOD*; Long integration time (~2 S)	Inhomogeneity of AgNPs size; Inconsistent surface roughness
		**λ** = 785 nm				
	[17]	Potentiostatic	WE: Au-capped Silicon nanopillars; CE: Pt; RE: Ag/AgCl	Melamine detection in milk	Cost-effective; High repeatability and sensitivity; Low *LOD*	Require complicated preparation process; Need pretreatment on the sample; Limited applicability to other analytes
		**λ** = 785 nm				
	[68]	CV, CA	WE: Screen-printed Carbon decorated with AgNPs; CE: Carbon disc RE: Ag wire	Reaction mechanism of resazurin/resorufin/dihydroresorufin system	Cost-effective; Dual monitoring strategy	High requirement on the compatibility between different techniques
		**λ** = 785 nm				
	[20]	CV	WE: Ag nanocube made 3D PLM; CE: Pt; RE: Ag/AgCl	Electrochemical reaction mechanism of [Ru(NH_3_)_6_]^3+^ and toxin methylene blue	“Mobile” SERS-active substrate; Excellent reproducibility; Smallest SEC cell; High sensitivity	Limited potential window; Complicated manufacturing processes
		**λ** = 532 nm				

NMR SEC	[69]	CV, CA	WE: Au thin film (50 nm) CE: Pt wire; RE: Pt foil (thickness, ~100 µm)	Redox behaviors of 1,4-Benzoquinone	High resolution; Potential dependent NMR characterization	High electrochemical cell resistance; Elaborate combining process between an electrochemical cell with an NMR tube
		500 MHz				
	[11]	CV,	WE: Polyaniline (PAn) coated ITO; CE: Pt wire; RE: Ag wire	Electro-catalysis of Hydroquinone	High applicability under varied experimental conditions (such as solvent composition, pH values, etc.)	Non-potential dependent electrolysis. Relatively low sensitivity of NMR caused by limited diffusion
		500 MHz				
	[70]	CV, CA	WE: Pt/MoS_2_/GNS coated ITO; CE: Pt wire; RE: Saturated calomel	Reaction mechanism of EOR	Real-time measurement; In situ NMR set up; Good compatibility	A relatively complex preparation process for composite material
		500 MHz				
	[71]	CV, CA	WE: Carbon fiber; CE: Pt wire; RE: Ag/AgCl	Electroreduction process of *p*-benzoquinone	High resolution; Enhanced electron reduction rate of *p*-benzoquinone	Limited electrochemical application to other analytes
		600 MHz				

DFM SEC	[14]	CV	WE: AgNPs modified Pt_90_Ir_10_ alloy wire; CE: Pt wire; RE: Ag/AgCl	Redox reactions of AgNPs in KCl solution	Realization of real-time visualized video streaming of the oxidation processes	High requirements on the compatibility between different techniques
		Halogen lamp				
	[72]	CV	WE: ITO; CE: Pt wire; RE: Pt wire	Influence of halide anion (F^−^, Cl^−^, Br^−^) on localized surface plasmon resonance of AuNRs	Comprehensive analysis of resonance energy, line width, and intensity of AuNR plasmon on individual entity level	Limited options for choosing different WEs
		--				
	[73]	CV	WE: AuNPs modified ITO ultramicroelectrode; CE: Pt wire; RE: Ag/AgCl	The oxidation process of Hydrazine	A better understanding of catalytic reactions and reproducibility	Broad DFM signal distribution; High homogeneous requirement on ITO surface to eliminate bad local contact and contribute to DFM’s background signal
		Halogen lamp or 632.8 nm laser				
	[74]	CV, LSV	WE: Ag nanocubes modified ITO; CE: Pt wire; RE: Pt wire	Deposition mechanism of copper on individual Ag nanocube	Can simultaneously track multiple NPs; Direct observation of formation kinetics and morphology on a nanoscale level	Laborious; Confined to OTE; Maintain enough interparticle distance to eliminate reactant diffusion
		Halogen lamp				

CV: Cyclic voltammetry; CA: Chronoamperometry; LSV: Linear sweep voltammetry; **λ**: laser wavelength; EDOT: 3,4-ethylenedioxythiophene; CAT/DA: catechol/dopamine; DA/EP: dopamine/epinephrine; *LOD*: Limit of detection; OER: Oxygen evolution reaction; 3D: three-dimensional; PLM: plasmonic liquid marble. EOR: ethanol oxidation reaction; MoS_2_: ethanol oxidation reaction; MoS_2_: molybdenum disulfide; GNS: graphene nanosheets; GNS: graphene nanosheets. Pt_90_Ir_10_: The wire consists of 90% Pt and 10% iridium.

## 3. Surface-Enhanced Raman Spectroscopy SEC (SERS SEC)

As one of the two most used SEC setups, Raman SEC has been widely used in various research fields [24,75,76]. It is well known that Raman spectroscopy is a powerful technique widely used to study the material structure because of its convenience, low price, and non-destructive characteristics [77]. However, Raman scattering is an inelastic scattering process with an external cross-section. Hence, Raman has limited sensitivity and consequently constrained analysis efficiency and applicability [5].

### 3.1. Nanostructure-Defined SERS-Active Substrates

**Figure 4:**
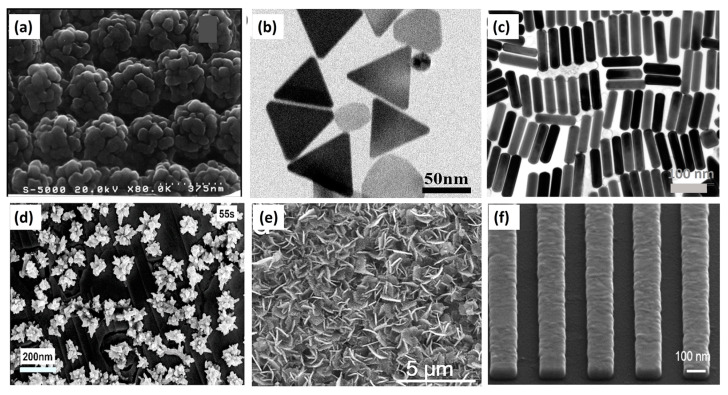
(**a**) SEM image of an ordered hollow Au/Ag nanostructured film. Reprinted from [89], with permission from ACS Publications. (**b**) TEM image of Ag triangular nanoplates. Reprinted from [90], with permission from Elsevier. (**c**) TEM image of Au nanorods (AuNRs). Reprinted from [91], with permission from ACS Publications. (**d**) SEM image of Ag nanoflowers (AgNFs)/Graphene/Copper (Cu). Reprinted from [92], with permission from Elsevier. (**e**) SEM image of Ag/WO_3-x_ nanoflakes. Reprinted from [93], with permission from Elsevier. (**f**) SEM image of Au nanostripes. Reproduced under the terms of CC BY licensed from [94].

Surface-enhanced Raman spectroscopy (SERS) was introduced in 1974 by Fleishman et al. to solve the above problems [78]. The SERS enhancement factor of the Raman signal can be as high as 10^15^ [79]. In SERS, the Raman substrate is a rough or nanostructured noble metal surface [67,68,69,70,71,80,81]. Under proper incident light, this metal surface will give rise to enhanced local electromagnetic fields via the localized surface plasmon resonance effect [5]. Up to now, considerable work has been done on the design of ideal SERS-active substrates [82,83,84]. SERS-active substrates in SEC setups include electrodes roughened by the oxidation–reduction cycle [24], metal island films [85], colloidal NPs, and surface-confined nanostructures [86,87,88]. There are many types of different SERS-active substrates, either as structural motifs or as SERS materials, as shown in Figure 4.

### 3.2. Applications of SERS SEC

The combination of SERS with electrochemistry has emerged as a powerful tool to monitor the structural changes of surface adsorbates [95,96], reaction intermediates [97,98], and quantitative analysis of electrolysis products [24,60]. Daniel and co-workers showed that the SERS SEC technique achieved a theoretical ferricyanide detection limit (~1.5 × 10^−8^ M) in a 0.1 M KCl solution [24]. This was well below the limits of traditional electrochemical measurements (1 × 10^−4^ M). In this work, one commercially available silver screen-printed electrode (SPE) was applied, and the rough WE surface decorated with AgNPs was obtained via the in situ electrochemical activation strategy. More details about the SERS SEC setup are summarized in Table 2. Daniel et al. mainly conducted two experiments using the SERS SEC setup [2,24]. (i) To record the time-resolved SERS SEC of the Ferri/ferrocyanide electrochemical process. For this, they did a cyclic voltammetry (CV) experiment between +0.5 to −0.4 V at 0.05 V/s and recorded the Raman spectra every 1s. This experiment showed the correlation between the Raman response and the electrochemical transformation of the redox couple. This was mainly done to demonstrate the performance of the SERS SEC instrument. (ii) The use of in situ electrochemically activated Ag SPEs for the detection of ferricyanide and [Ru(bpy)_3_]^2+^. In this experiment, the electrochemical process also activated the Ag SERS-active substrate. They detected ferricyanide in concentrations as low as 1.5 × 10^−8^ M in a 0.1 M KCl solution and [Ru(bpy)_3_]^2+^ as low as 2.1 × 10^−8^ M in a 0.1 M KCl solution. This result demonstrated the potential of SERS SEC for the sensitive, precise, and rapid detection of different analytes. However, using this typical oxidation–reduction cycles method to fabricate a SERS-active substrate often has the problem of inconsistent surface roughness and results in low reproducibility issues [20].

**Figure 5:**
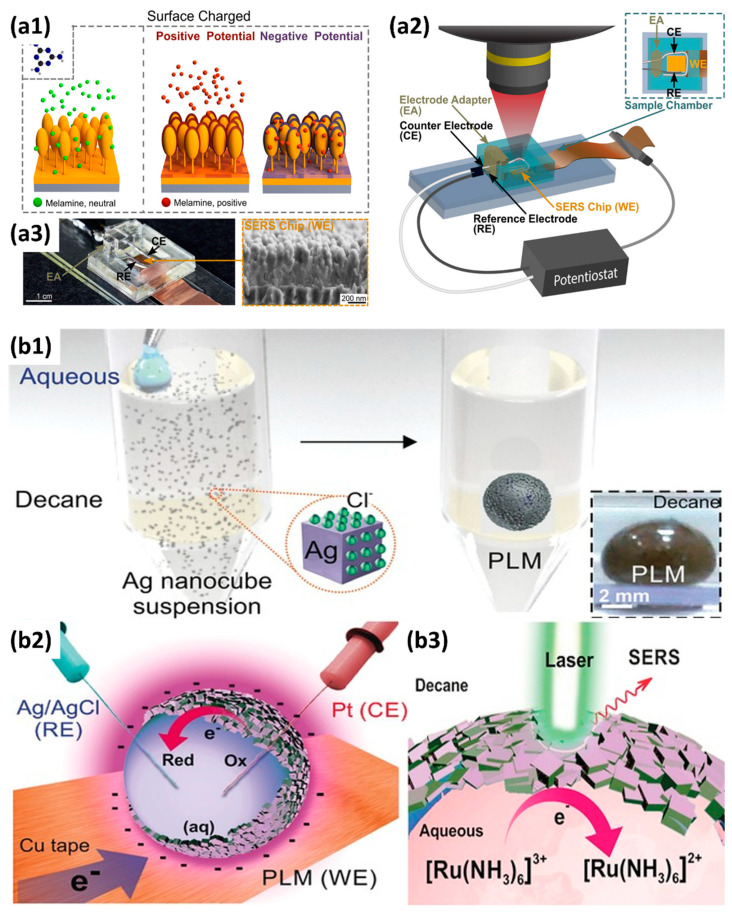
(**a1**) Schematic diagram of the electrochemically assisted SERS-based detection working principle. (**a2**) Illustration of the custom-made electrochemical-SERS platform and its respective system interfacing. (**a3**) Photo of the assembled detection chamber and SEM image of Au-capped nanopillar structures for SERS detection. Reprinted from [17], with permission from ACS Publications. (**b1**) Fabrication of PLM. Inset: digital image of PLM. (**b2**) Using the Ag shell as a 3D working electrode (WE). (**b3**) Electrochemistry-SERS investigation using PLM. Reprinted from [20], with permission from John Wiley and Sons.

For SERS-based analytical transducers, reusability is critical for decreasing the variation between measurements and the manufacturing time. Marlitt et al. proposed one possible way to prepare a standard/practical analytical tool with excellent reusability [17]. The structure of the SERS SEC setup is shown in Figure 5(a1–a3). In this setup, the forest of Au-capped silicon nanopillars was applied as the SERS-active substrate and the WE. The detection of the toxic compound melamine was carried out. Instead of using harsh reagents [17], UV irradiation [99], or high-temperature treatment [100], the active substrates’ recycling was obtained by applying a small positive voltage (+0.8 V, 1 min). The electrostatic force could successfully remove the positively charged melamine to refresh the substrate (relative standard deviation < 11.4%). Finally, an *LOD* of 0.01 ppm in PBS and an *LOD* of 0.3 ppm in milk were obtained, which were low enough for the established maximum allowed levels (1 ppm) in powdered infant formula.

Except for substrates with defined nanostructure morphologies (such as NPs, nanopillar forests, and nanodot arrays, among others), suspended metallic NPs have been used as “mobile” SERS-active substrates, especially in microfluidic devices [101,102,103]. Recently, Ling et al. reported using plasmonic liquid marbles (PLMs) in SERS SEC, as shown in Figure 5(b1–b3) [20]. In this work, the three-dimensional (3D) PLM covered by a shell of Ag nanocubes (Ag @ PLM, Ag nanocubes’ average edge length is around 133 ± 9 nm) was prepared as a lab-on-a-droplet microliter-scale SEC cell. Ag @ PLM was exploited as a bifunctional SERS platform and concurrently as a WE for redox process modulation. Remarkably, the PLM’s synergistic electrochemical and SERS capability elucidated critical insights into the electrochemical reaction mechanism and molecular structural changes of ruthenium hexammine (III) chloride and toxin methylene blue. Finally, this novel 3D SERS SEC cell exhibited two-fold and ten-fold better electrochemical- and SERS activities than conventional 2D counterparts. However, the application of such kind of “mobile” SERS substrates can cause some problems in actual use, which we should also keep in mind: (i) contamination and clogging issues [104]; (ii) interference with the other downstream bio/chemical processes; and (iii) poor reproducibility, which is due to batch-to-batch variances of NP synthesis as well as aging of the colloidal suspensions [105,106].

## 4. Nuclear Magnetic Resonance SEC (NMR SEC)

When studying electrochemical systems, it would be beneficial to obtain (either concentration or structural) information on the reaction reagents, intermediates, and products during the electrochemical reactions to determine the possible reaction pathways. Among the spectroscopy techniques, nuclear magnetic resonance (NMR) spectroscopy is one of the frequently used techniques to elucidate the molecular structures of target analytes. Meanwhile, it is well suited for coupling with in situ electrochemical techniques [107]. It typically operates within the radio frequencies of 60 to 100 MHz. These low-energy waves can interact with nuclei with magnetic spins, such as isotopes ^1^H, ^15^N, and ^13^C. For NMR SEC, the different spin states of nuclei become separated with a powerful magnetic field. The surrounding atoms and functional groups in a molecule influence how strongly the outside magnetic field affects the target nucleus locally. Consequently, NMR SEC can obtain comprehensive structural information on the molecules. NMR SEC has investigated electrocatalytic processes [108,109], reaction intermediates [110,111], reagents, and product concentrations [112,113,114,115]. NMR SEC has been used to model the redox reaction processes of different analytes (such as hydroquinone (QH_2_) and phenacetin), as summarized in Table 2 [116,117].

Although the NMR SEC technology has been dramatically improved and has shown immense potential, compared with the above UV-Vis SEC and SERS SEC techniques, NMR SEC has been limited to a few specialized groups since no commercial NMR SEC cells can be easily assembled for routine measurements [78]. Richards et al. did pioneering work combining NMR with in situ electrolysis or NMR SEC in 1975 [118]. In Richards et al.’s seminal work, the flow cell and NMR tube were integrated into a two-electrode NMR SEC cell. A mercury (Hg)-coated Pt wire was used as the WE, and an uncoated Pt wire was used as a CE, as depicted in Table 3. Electrolysis products of *trans*-1-phenyl-1-buten-3-one (C_6_H_5_CHCHCOCH_3_) were released into the detection region from the exit capillary (flow rate ≥ 0.2 mL/min). The successful observation of the reduction of C_6_H_5_CHCHCOCH_3_ to 1-phenyl-3-butanone (C_6_H_5_CH_2_CH_2_COCH_3_) in the alkaline environment validated the successful combination of NMR with electrochemistry.

### 4.1. Deteriorations of Magnetic Field in NMR SEC

However, in most of the proposed electrochemical NMR cells, the electrodes are placed inside the NMR coil, which will deteriorate the magnetic field homogeneity and reduce the signal-to-noise ratio. The conducting metallic electrodes disrupt the homogeneity of the magnetic field, a critical requirement for NMR [119,120]. Thus, NMR SEC is more complicated than other SECs [116]. A few research groups have made great efforts to address the electrode structure’s problem and reduce or eliminate the disruption in the homogeneous magnetic field caused by the electrodes [11,121,122]. One detailed summary of the NMR SEC’s development is in Table 3. This table summarizes some representative work and shows the electrochemical cell design with the modified electrode structure. The continual optimization of NMR SEC electrochemical cells and the usage of new electrode materials has led to many novel studies of NMR SECs. The main approaches to overcome the challenges include:

(i) Placing the electrodes outside the detection region [117,123,124];
(ii) Using secondary coils [107] or radiofrequency chokes [122,123,125,126];
(iii) Using thin film metallic electrodes [121,127,128];
(iv) Using nonmetallic electrodes such as carbon microfibers and polymer electrodes [11,122,129].

### 4.2. Applications of NMR SEC

#### 4.2.1. NMR SEC for Ethanol Oxidation Reaction Application (Regular Electrode Configuration)

**Figure 6:**
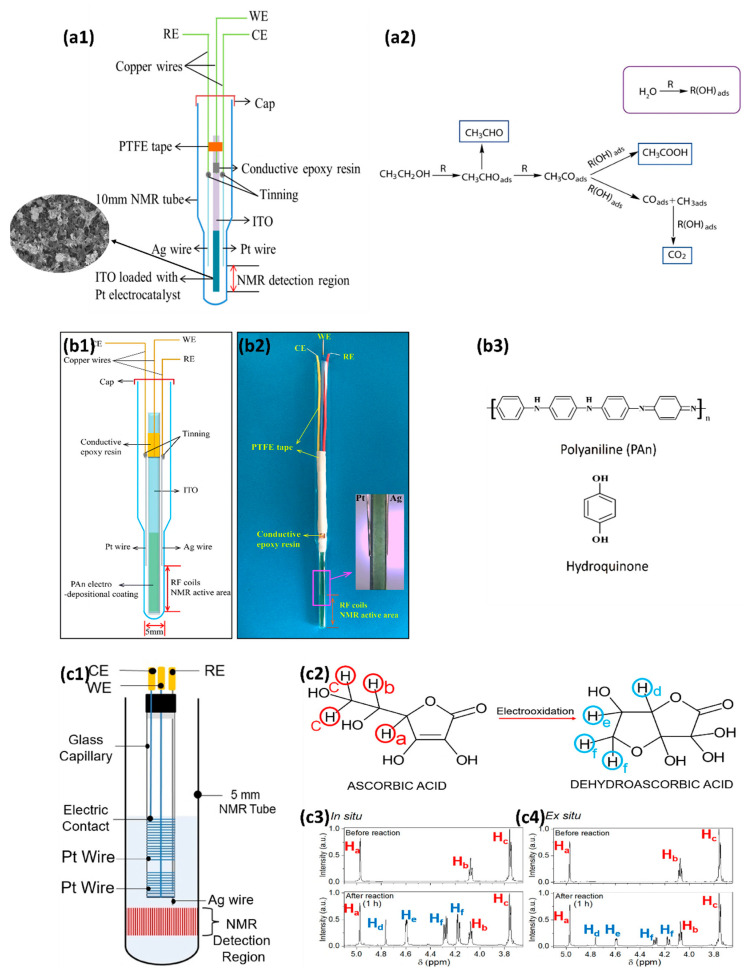
(**a1**) Schematic structure of the electrochemical cell designed for in situ EC-NMR experiment. (**a2**) Reaction mechanism of ethanol oxidation on Pt/MoS_2_/GNS. Reprinted from [77], with permission from Elsevier. (**b1**,**b2**) Electrochemical cell designed for in situ EC-NMR; (**b3**) The molecular structure of polyaniline (PAn) and hydroquinone (QH_2_). Reprinted from [11], with permission from Elsevier. (**c1**) Electrochemical cell and electrodes. The WE, CE, and RE are fixated on the glass capillary tube. (**c2**) The different hydrogen atoms observed in the NMR spectra are highlighted. In addition, the 1H NMR spectra of the oxidation of ascorbic acid in situ (**c3**) and ex situ (**c4**) are shown. Reprinted from [78], with permission from Elsevier.

Direct ethanol fuel cells have aroused tremendous research interest because of their high energy density, environmental friendliness, easy refueling, and low operating temperatures [130,131]. Therefore, to monitor molecular changes of reaction products and unveil the reaction mechanism of the ethanol oxidation reaction (EOR), Wang et al. introduced the in situ real-time setup of electrochemical NMR (EC-NMR), as shown in Figure 6(a1), in which a Pt wire is serving as the CE, and an Ag wire is serving as the RE [77]. An ITO electrode decorated using hybrid materials of small-sized (~5.4 nm) PtNPs supported on molybdenum disulfide combined with graphene nanosheets is used as the WE. Employing in situ NMR, molecular information of the products and reactants is studied simultaneously during the electrochemical process. It successfully fulfills the purpose of elucidating the reaction mechanism of EOR, as shown in Figure 6(a2).

#### 4.2.2. NMR SEC for QH_2_ Application (Using Polymer Electrode)

As we mentioned above, to minimize the interference to the magnetic field homogeneity and obtain a high signal-to-noise ratio, the recent use of conductive polymer polyaniline (PAn) to form ITO/PAn composite WE in NMR SEC has attracted wide attention [11]. The high conductivity, good redox reversibility, and excellent environmental stability of PAn have made it a material of choice in electrocatalysis [132]. Figure 6(b1,b2) describe the EC-NMR cell that uses ITO/PAn to composite the WE. The device was used to monitor the oxidation process of hydroquinone (QH_2_) for the first time (Figure 6(b3)). The high sensitivity of the NMR SEC technique allows the authors to monitor the generation of products quantitatively and precisely under varied solvent composition ratios and pH values.

#### 4.2.3. NMR SEC for Ascorbic Acid Application (Using Magnetohydrodynamic Effect)

As electrodes or flow cells, ultra-thin metallic films require complex fabrication protocols. In addition, nonmetallic electrodes usually have limited electrochemical applications due to the low achievable currents [78]. To avoid these limitations, one frequently used methodology is to place the metallic electrode above the NMR detection area, as shown in Figure 6(c1). Here, one interesting point is introducing the magnetohydrodynamic (MHD) effect instead of simply placing the electrode above the NMR detection area. The main force acting to create this effect is the magnetic force, which results from the cross-product between the ionic current density and the external magnetic field [133,134]. The stirring force generated by the MHD effect can perfectly homogenize the reagent and product concentration in the detection region, allowing the NMR to sense the analytes in real time.

**Table 3:** A summary of NMR SEC’s development history.

Publish Time	1975	2000	2009	2018
**Ref.**	[118]	[121]	[122]	[11]
**Electrode System**	**WE**: Hg-coated Pt wire **CE**: Pt (Wire)	**WE**: Tubular Au film **CE**: Cylindrical Pt-mesh **RE**: Ag/AgCl	**WE**: Carbon fiber filament **CE**: Carbon fiber filament **RE**: Thin chlorinated Ag wire	**WE**: Polyaniline (PAn) coated ITO **CE**: Pt (Wire) **RE**: Ag wire
**Cell structure**	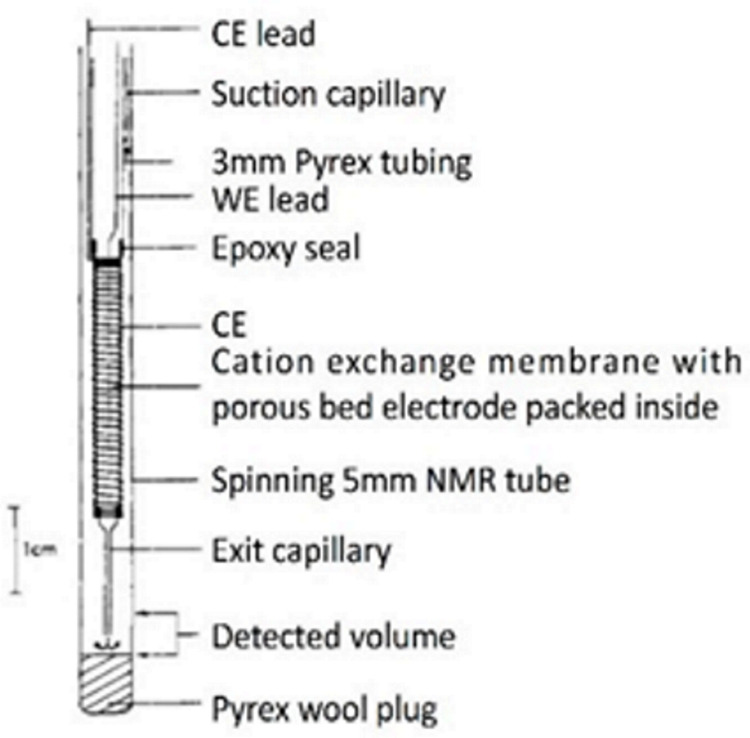	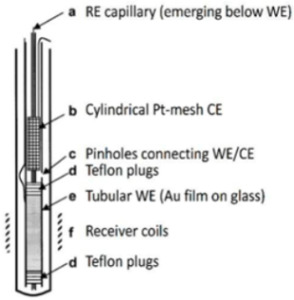	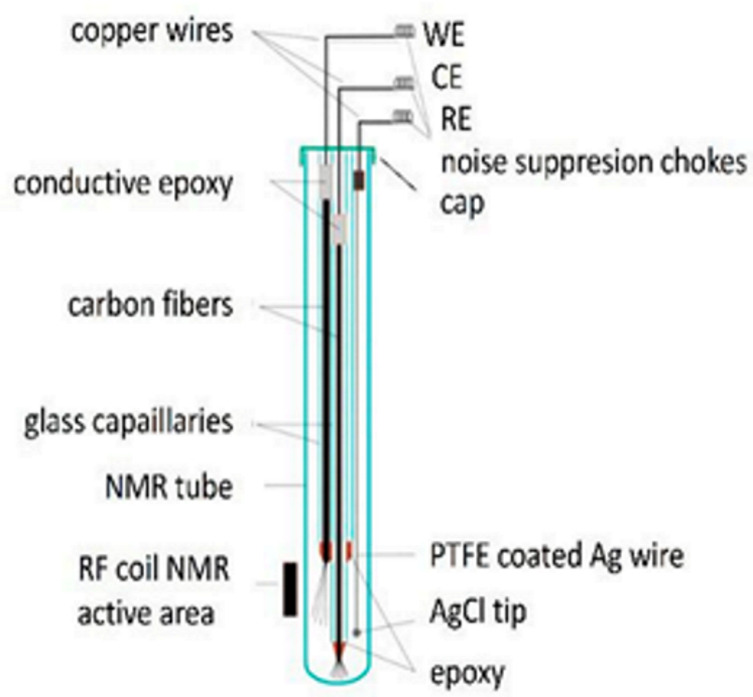	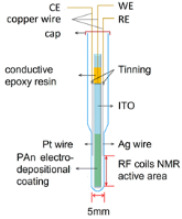
**System Studied**	Reduction of trans-1-phenyl-1-buten-3-one	Reduction of *p*-benzoquinone	Reduction of *p*-benzoquinone	Oxidation of hydroquinone
**Advantages**	Without the need to modify the NMR probe	Minimal influence on homogeneity magnetic field; Unmodified probe and outstanding resolution and sensitivity	Easy to prepare and broad applicability, suitable for a large potential window	Fast and with the capability of quantitatively monitoring the generation of products under varied solvent compositions and pH values
**Drawbacks**	Low resolution, line broadening, and toxic metal are used	Slow diffusion from the inactive region	Take a long time for results to be ready (~6 h)	The probe requires a relatively complex preparation process.

As shown in Figure 6(c1), Silva et al. used Pt wires to prepare the WE, CE, and Ag wires for making the RE. The electrodes were fixated on a capillary glass tube, inserted into a standard 5 mm NMR tube, and placed 0.5 mm above the NMR detection region. The in situ observation from AA to dehydroascorbic acid (DA) was successfully achieved (Figure 6(c2–c4)). Compared to the ex situ configuration, a two-time more significant conversion efficiency of the electrooxidation from AA to DA was observed.

## 5. Dark-Field Microscopy SEC (DFM SEC)

### 5.1. Localized Surface Plasmon Resonance (LSPR) in DFM SEC

**Figure 7:**
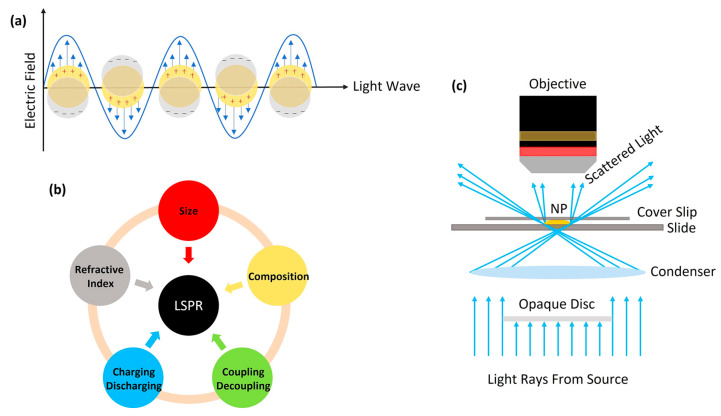
(**a**) Schematic illustration of the LSPR of a metallic and spherical nanoparticle. (**b**) The main influencing factors and adjustment strategies on LSPR of the plasmonic NPs. (**c**) Schematic diagram of one dark-field microscope.

Nowadays, metallic NPs such as AuNPs, AgNPs, and GeNPs have emerged as a class of materials with unique optical [31,135,136], catalytic [137,138], mechanical [139,140], and biological properties [31,141,142,143]. AgNPs have been widely used in customer products (such as phones, refrigerators, etc.) and medicine due to their exceptional antibacterial and anti-inflammatory effects [144,145]. AuNPs have been used to detect bacteria [146], solar cells [147], and ionic electrolytes [148]. A representative illumination of an NP’s localized surface plasmon resonance (LSPR) is shown in Figure 7a. The confined electrons in the conduction band, the electron cloud, are displaced by an incoming electric field (light) [149]. The negatively charged electron cloud is withdrawn again due to the Coulomb Forces of the remaining fixed and positively charged nuclei.

Electron-rich metal NPs, such as AuNPs, AgNPs, and PtNPs, can exhibit an intrinsic LSPR, which is defined by the particle size, shape, composition, interparticle spacing, and dielectric properties of the local environment that surrounds the particles (Figure 7b) [14]. Therefore, an NP’s oxidizing/reducing in aqueous suspensions will lead to a shift in the LSPR frequency. Based on this property, the NP’s chemical state can be traced and analyzed by probing the changes in the spectral extinction spectra. However, such information cannot be accessed by the established non-spectrally resolved optical methods, which only monitor the signal intensity [150]. Furthermore, it is well demonstrated that the redox potential of NPs is size-dependent [151,152,153]. The synthesis protocols for NPs will usually lead to an inherent heterogeneity in the NP size distribution [154,155]. Therefore, studies that can look at a single NP within a heterogeneous NP distribution will allow researchers to observe trends that are not possible through existing ensemble electrochemical techniques.

### 5.2. Applications of DFM SEC

The dark-field scattering technique is receiving more and more attention since the ability to reveal changes at a single-entity/nanoscale level is not readily accessible by the established in situ measurements such as fluorescence spectroscopy and SERS [14]. Dark-field microscopy (DFM), in conjunction with electrochemical techniques (hereafter, DFM SEC), allows direct observation of the chemical reactions occurring on a single NP. In Figure 7c, the light is scattered by the NPs on the slide. The NPs on the slide are thus brilliantly illuminated against the dark background. Then the LSPR is tracked by scattering in the near-infrared region [60,156,157,158,159]. The LSPR extinction maximum of the NP can be measured with DFM and recorded using an electron multiplying charge-coupled device (EMCCD) camera. Consequently, DFM SEC is applied to study the oxidation processes on a single NP.

**Figure 8:**
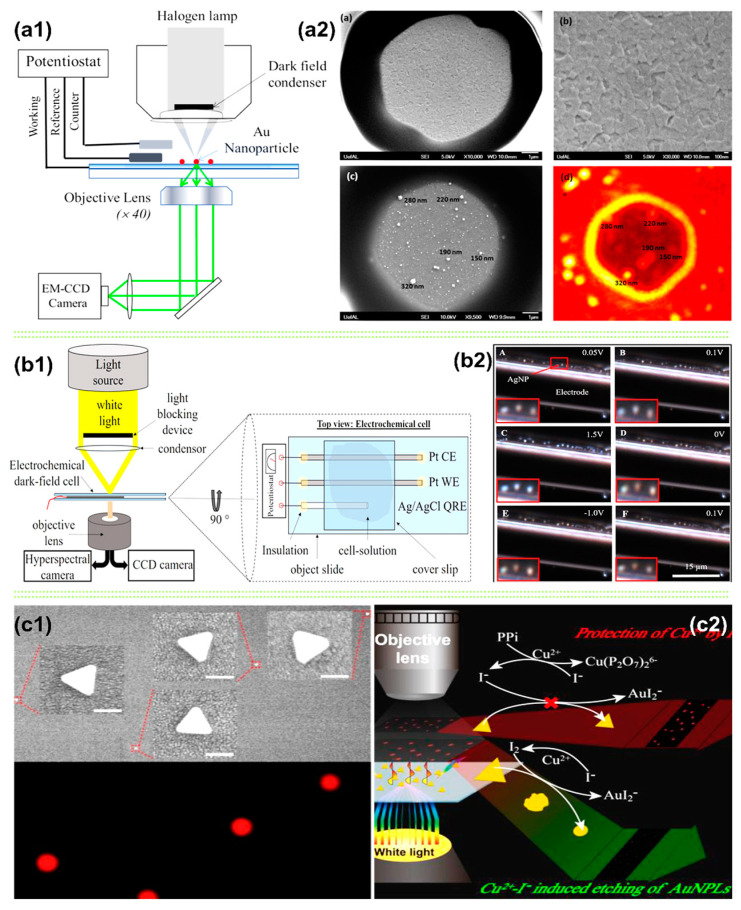
(**a1**) Experimental schematic of the DFS-EC setup for hydrazine oxidation. (**a2**) SEM images of a bare ITO ultramicroelectrode (top two images). Reprinted from [80], with permission from ACS Publications. SEM and DFS images of AuNPs’ modified ITO electrode (bottom two images). (**b1**) Experimental setup of the dark-field microscope and the used electrochemical cell; (**b2**) DFM CCD images recorded during CV at selected potentials; exposure time: 70 ms. Reprinted from [14], with permission from ACS Publications. (**c1**) The co-localization images of the DFM and SEM of Au triangular nanoplates (AuNPLs). (**c2**) Working principle of single-nanoparticle analysis (SNA) for PPi assay. Reprinted from [161], with permission from ACS Publications.

Nanostructured electron-rich metals such as Au, Ag, and Pt exhibit strong light-scattering and absorption characteristics when their surface electrons are optically excited under resonance conditions from surface plasmons [80,159,160,161,162]. AuNPs have been widely used in clean energy transformations due to their excellent and stable catalytic efficiency [80]. In this paper, shown in Figure 8(a1,a2), Pan’s group used hydrazine as a model to study the local catalytic activities and structure-functionality relationships at a single AuNP level. The kinetics of electrocatalytic oxidation of hydrazine at AuNPs were analyzed in real time using the light-scattering SEC method at planar and miniaturized ITO electrodes. Compared with the NP detection method based on spontaneous collision events, the ITO ultramicroelectrode technique and DFS provided a better understanding of the catalytic reactions and their reproducibility.

Recently, Kevin et al. studied real-time in situ oxidation of a single AgNP (~50 nm) in the presence of Cl^−^ ions using DFM SEC. Figure 8(b1) is Kevin et al.’s DFM SEC device schematic. Hyperspectral imaging (HSI) and a CCD camera were used to record the changes in spectral position and LSPR intensity during the CV (50 mV/s) electrochemical process. Figure 8(b2) gives the CCD snapshots of individual AgNPs under different applied potentials during the CV. This allowed the researchers to observe and analyze the redox process of AgNPs in the presence of Cl^−^. Upon applying a ~0.1V potential, the AgNPs oxidized to Ag chloride (AgCl). The AgCl continued to oxidize to Ag_2_O_3_ or AgClO_2_ with an increase in potential (~1 V). During reverse scanning (a decrease in the potential down to ~0 V), the oxides were reduced to AgNP. It is worthwhile to note that this work also provided a comprehensive microparticle characterization method.

In addition to different NPs, different nanostructured metals are used in the DFM SEC technique. The excellent LSPR characteristics and high anisotropy make Au triangular nanoplates (AuTNPs) stand out due to their sharp vertices, providing electric-field-enhanced hotspots [160,161]. A more recent experimental work by Gu et al. shows the successful use of AuTNPs to monitor the pyrophosphate (PPi) sensitively and selectively [160,163]. This critical biological anion plays significant roles in various fundamental physiological processes (such as cellular metabolism and RNA and DNA polymerizations) [164,165,166]. This work was based on the inhabitation effects of PPi against the etching of AuNPLs in the Cu^2+^ and I^−^ ions solution. The etching of AuNPLs by the Cu^2+^ and I^−^ ions lead to a blue shift and intensity decrease in the LSPR scattering spectra of AuNPLs. However, adding PPi can prohibit the etching of AuNPLs due to the strong affinity of PPi to Cu^2+^ ions. Based on these facts, Gu et al. successfully established a simple, sensitive, and selective single-particle analysis platform for quantitatively detecting PPi, even for real biological samples.

## 6. SEC Techniques’ Applications in Microfluidics

It is well known that the often-cited advantages of microfluidics, including faster response times, lower reagent volumes, and potential for integration, are significant considerations in the research work [167]. After studying the recent developments of SEC techniques, one interesting fact is that the combination of SEC and microfluidics is becoming a trend in research papers [104,168]. However, due to the limited use of the DFM technique and the confined cell structure of NMR, the present applications mainly rely on the combination of SERS/Raman- or UV-Vis-based SEC + microfluidics.

### 6.1. Applications of SERS/Raman SEC in Microfluidics

**Figure 9:**
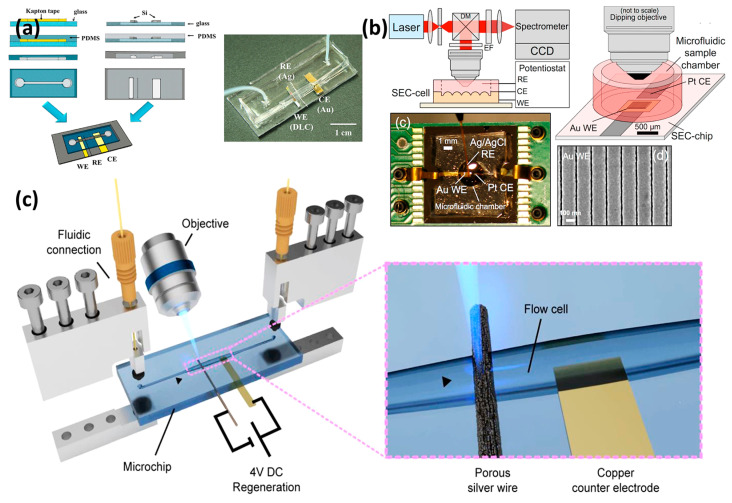
(**a**) Fabrication steps for a three-electrode microfluidic device. Right: The process to produce a PDMS microfluidic device with a three-electrode configuration. Left: As-assembled microfluidic device with embedded electrodes. Reprinted from [169], with permission from ACS Publications. (**b**) In situ SERS SEC analysis system. Reprinted from [93], with permission from ACS Publications. (**c**) Illustration of the microfluidic setup for SERS measurements. Reprinted from [104], with permission from ACS Publications.

Based on limited available papers, a few representative studies done with the integrated techniques are shown in Figure 9. One relatively straightforward but attractive configuration is proposed by Singh et al. for the highly sensitive detection of okadaic acid (OA) [168]. In this combined detection module, the microfluidic chip was employed to mix OA and the OA aptamer well. The phosphorene–gold nanocomposite-modified screen-printed carbon electrode (SPCE), which posed an affinity to the OA aptamer, was subsequently analyzed. The high performance of OA detection, whether qualitative or quantitative, demonstrated that the proposed point-of-care device can be deployed to perform on-farm assays in fishing units.

“Immobile” SERS-active substrate means nanostructures with defined morphology (such as NPs, nanopillar forests, and nanodot arrays, among others) are permanently attached to substrates. For example, in the recent work published by Triroj et al., a diamond-like carbon thin film was prepared as a biosensing platform/substrate in the microfluidic device, as shown in Figure 9a [169]. An in situ microfluidic analysis system is reported by Yuan et al. using nanostructured Au surfaces as the WE and simultaneously SERS-active substrate [93]. Information about the microfluidic device and nanostructured Au substrate can be found in Figure 9b. However, a drawback of “Immobile” SERS substrates is that they are intended for one-time use only [170]. With the pursuit of repeating “Immobile” SERS, Belder et al. successfully fulfilled the regeneration of the SERS substrate by applying pulsed voltages, which had been demonstrated with high reproducibility. This work incorporated the chemically roughened silver wire into the microfluidic chip and it was used for SERS measurements. The electrical regeneration process for the silver wire SERS substrate by applying a potential to clean the SERS substrate was achieved based on the proposed structure (Figure 9b). Furthermore, the high reproducibility of Malachite green’s Raman spectra confirmed the achievement for the purpose of multiple recycling of the same SERS substrate.

### 6.2. Applications of UV-Vis SEC in Microfluidics

**Figure 10:**
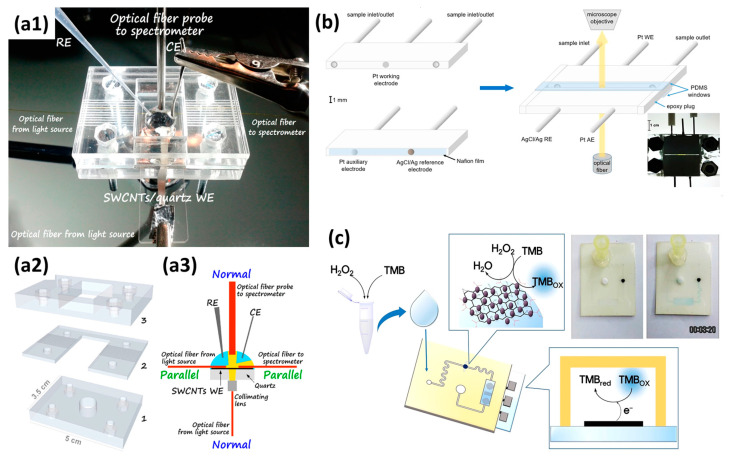
(**a1**) a Photograph of the assembled cell ready to measure, (**a2**) a schematic view of the disassembled cell, and (**a3**) a detailed schematic view of the experimental setup. Reprinted from [174], with permission from ACS Publications. (**b**) Illustration of the microfluidic setup for UV-Vis SEC measurements. Reprinted from [175], with permission from Elsevier. (**c**) A schematic diagram of H_2_O_2_ detection on electrochemical POC devices with Au@PtNP/GO nanozymes. Reprinted from [172], with permission from Elsevier.

Similarly, the combined methodology of UV-Vis SEC and microfluidics has been widely used in biotechnology, catalysis, environmental protection, and others [7,171,172,173,174]. However, compared with the SERS/Raman SEC, the employment of UV-Vis SEC in microfluidics is more used, which is probably because the electrode substrate can be more easily prepared. As shown in Figure 10(a1–a3), in this interesting work reported by Colina et al., one easy method to employ or transfer commercial SWCNTs to different nonconductor and transparent supports as the WE is reported [174]. This work removes the often-employed hydraulic press step from the WE preparation process, significantly expanding the possibility of transferring the SWCNT film to almost any support. Another interesting point of this work is the employment of bidimensional SEC technology. As shown in Figure 10(a3), two different light beam arrangements, namely, normal and parallel transmission arrangements, are integrated into the same device to collect complementary information during the ferrocenemethanol electrode processes. Another interesting microfluidic device for UV-Vis SEC is proposed by Wang et al. [175]. A parallel transmission arrangement was adopted in this paper, which avoided the OTEs. Spectral measurements were made using an “in-house” constructed visible micro spectrometer which consisted of a deuterium/tungsten–halogen light source and a CCD spectrometer.

In the work shown in Figure 10b, Seong et al. first reported one electrochemical point-of-care device with nanozymes for the high quantification of hydrogen peroxide (H_2_O_2_), a molecule for signaling within cells [172]. The electrodes (WE, CE, RE) were prepared using the market-available ITO electrodes. Then, as depicted in Figure 10b, the artificial nanostructured enzymes were immobilized in the microfluidics channel, showing a robust catalytic activity toward 3,3′,5,5′-tetramethylbenzidine (TMB) substrate in the presence of H_2_O_2_. The oxidized TMB with a blue color was subsequently analyzed using the UV-Vis SEC technique. Finally, based on the proposed device structure, a broad detection of H_2_O_2_ ranging from 1 µM to 3 mM and a low *LOD* of 1.62 µM were successfully obtained.

## 7. Summary & Outlook

We have detailed the recent developments in composite SEC techniques, including UV-Vis SEC, Raman SEC, DFM SEC, NMR SEC, and recent progress in combining SEC techniques and microfluidics. In addition, a detailed analysis of the working principle and problems encountered in the selected applications are summarized. As mentioned above, the combination of electrochemistry and spectroscopy (SEC techniques) has been applied to diverse research fields ranging from the electron transfer process [55,176], reaction mechanisms [167], forensics sciences [177], and determination of intermediates and final products in electrochemical reactions [112,113]. Furthermore, the continuous advancement in nanotechnologies and the use of new materials (NPs [20,77], conductive polymers [11], and composite materials [17,178]) have further promoted SEC techniques. However, each of the SEC techniques mentioned above is still suffering some limitations, from the lab-scale to widespread practical use, as summarized below:

UV-Vis SEC—For the UV-Vis SEC technique, as shown in Figure 2, OTEs are almost an inevitable topic in the normal transmission arrangement. (i) However, the frequently used OTEs such as ITO and FTO have the issue of fewer negative inert potential windows, and the thin film metallic OTEs are limited to electrochemical studies requiring high potentials due to the corresponding metal oxidation [50]. Therefore, considering the limitations of OTEs, more and more people are choosing parallel arrangement configurations, as summarized in Table 2. Compared with normal transmission arrangements, the parallel arrangement configuration is more favorable for conducting bidimensional SEC techniques [10]. (ii) However, in the parallel working mode, a perfect but difficult alignment of the light beams is required, complicating the operation process.

SERS SEC—For the SERS SEC technique, according to the latest statistics from the website of the web of science, compared with other SEC technologies, the Raman SEC technique is the most widely used one (Figure 1b). More and more combinations between SERS and electrochemistry have been used, considering the huge enhancement factor of the Raman signal [86]. Different metallic/composite NPs or other confined nanostructure morphologies such as nanopillar forests and nanodot arrays have been prepared and studied [101,102,103]. However, (i) the need for nanostructured SERS-active substrates will undoubtedly increase the experiment’s difficulty, cost, and time. (ii) An important issue is their reproducibility, considering the inherent batch-to-batch variances of NPs synthesis and the difficulty of storing. The other difficulties include (iii) background noise in the Raman signal and (iv) complicated instrumentation for the incorporation into a point-of-care or point-of-use system. Though handheld Raman spectroscopes exist, they are limited by their resolution and bandwidth. Hence, developing the Raman active SERS substrate will be a critical area of research for the broad application of this SERS SEC technique.

NMR SEC—For the NMR SEC technique, this seems to be the most versatile as a secondary technique for identifying the molecular signature of the captured chemical moieties or the small biomolecules. However, due to the low sensitivity issue of the NMR technique, most of the uses for NMR SEC are focused on collecting information on a reaction intermediate during the electrochemical process to determine the possible reaction pathways [77,78,107]. The limitations to the widespread use of NMR SEC are: (i) the deterioration of magnetic field homogeneity due to metallic conducting electrodes; and (ii) using thin metallic or nonmetallic electrodes such as carbon microfibers or polymer electrodes usually requires complex fabrication protocols. Furthermore, nonmetallic electrodes usually have limited electrochemical applications due to the low achievable currents.

DFM SEC—For the DFM SEC technique, unlike the other SEC techniques, this focuses more on the study between structural characteristics and catalyst activities from a single NP level. Since the understanding at the nanoscale level is critical to designing and producing stable and high-performance catalysts, however, by studying articles published in recent years (Figure 1b), we can see that before the widespread applications in the research community, there is a considerable gap for this technology to cross. The reasons are listed here: (i) this technology has high requirements for the electrode materials: OTEs are required in DFM SEC. (ii) Tedious coupling procedures of the light and electric paths. (iii) Further, DFM SEC setups require extensive optics and might not be easy to be incorporated into a point-of-care or point-of-use system. (iv) The reliability and device-to-device variation in DFM SEC are also concerns.

In summary, we have detailed the recent developments in composite SEC techniques, including UV-Vis SEC, SERS SEC, NMR SEC, and DFM SEC. A detailed analysis of their working principle challenges encountered in their applications and recent development directions are summarized. Using SEC techniques and microfluidics is becoming one interesting trend within research fields, ranging from biotechnology, catalysis, environmental protection, and others [7,171,172]. Of note, from the summary (Table 2), some articles employed the in situ bidimensional SEC methodology in their study, which will build a more comprehensive understanding of the reagent’s reaction mechanism, electron transfer mechanism, intermediates, the concentration of products, and relevant reaction pathway. Although there are many compatibility problems, mutual interferences, optical road layouts, and so on, this will be one of the promising development directions in the future. Although these SEC techniques have many limitations to break though, with the continual development of new functional materials and nanotechnology, people will finally solve these problems (such as the OTEs in UV-Vis/DFM SEC, reusability of SERS-active substrates in SERS SEC, or inhomogeneity of the magnetic field in NMR SEC) existing in SEC techniques. This review article will enable more people, either established researchers or novices, to become familiar with the use of SEC technology and ultimately achieve the goal of promoting SEC technology.

## Data Availability

Not applicable.
